# Ophthalmic signs in Ugandan adults with HIV-associated cryptococcal meningitis: A nested analysis of the ASTRO-CM cohort

**DOI:** 10.12688/wellcomeopenres.14666.2

**Published:** 2018-10-12

**Authors:** Rachel R Atherton, Jayne Ellis, Fiona V Cresswell, Joshua Rhein, David R Boulware

**Affiliations:** 1 Institute of Infectious Diseases, Kampala, Uganda; 2 Clinical Research Department, London School of Hygiene and Tropical Medicine, London, WC1E 7HT, UK; 3 University of Minnesota, Minneapolis, MN, USA

**Keywords:** Cryptococcus, cryptococcal meningitis, HIV, visual, ocular, ophthalmic

## Abstract

Cryptococcal meningitis is a leading cause of morbidity and mortality among HIV-infected persons, accounting for 15% of AIDS-related deaths. Visual disturbance is commonly reported, and a wide range of ophthalmic signs may be present on examination. There is limited published literature to date describing the range and incidence of ophthalmic signs in HIV-associated cryptococcal meningitis. Nested within the Adjunctive Sertraline for the Treatment of HIV-Associated Cryptococcal Meningitis (ASTRO-CM) trial (ClinicalTrials.gov number: NCT01802385), we conducted an observational study of 696 Ugandan adults with HIV-associated cryptococcal meningitis.

Patients were screened for visual disturbance and external ophthalmic signs at initial presentation and at follow-up appointments over 18 weeks. Assessment comprised simple clinical history and basic examination and required no specialist equipment.

More than a quarter of our cohort demonstrated ocular signs or symptoms, which were observed throughout the study period.  A broad range of ocular signs were demonstrated: these included neurological signs (10.9%), localized ocular pathology (4.5%), and evidence of concurrent systemic disease (12.9%).

The range of signs observed demonstrates the complexities of case management in patients with advanced HIV and cryptococcosis and also the importance of basic ocular examination in low resource settings.

There remains an urgent need for studies conducting comprehensive ocular examination in patients with HIV-associated cryptococcal meningitis; these studies should include formal assessment of visual acuity, slit lamp examination and dilated indirect ophthalmoscopy. Prospective studies should investigate whether there is a correlation between reported visual disturbance and objective signs, in order to further clarify the underlying mechanisms and to guide effective diagnosis, follow-up and management.

## Background

Cryptococcal meningitis is the commonest neurological complication in patients with advanced HIV, accounting for 15% of AIDS-related deaths ^1^. Visual disturbance is a frequent presenting symptom including reduced visual acuity, blurred vision, diplopia and photophobia. In a South African study of patients with HIV-associated meningitis due to *Cryptococcus neoformans*, 47% (40/86) had decreased visual acuity ^2^. Visual loss may occur secondary to raised intracranial pressure or be due to direct fungal invasion of the optic nerve, optic chiasm or optic tracts ^3, 4^.

Papilloedema is reported to be the most common ophthalmic sign in cryptococcal meningitis, prevalence ranging between 33% and 48% ^5– 7^ in case series. Other ophthalmic signs described in case reports include: retinal haemorrhages ^8^, multi-focal choroiditis ^9^, optic atrophy ^10^, vascular tortuosity ^11^, exudative retinal detachment ^12^ and intraocular cryptococcoma ^13^. Ophthalmic signs in patients with cryptococcal meningitis may be indicative of neurological dysfunction including cranial nerve palsies ^14, 15^ or may reflect concurrent systemic disease in patients with advanced HIV.

We conducted an observational study of HIV-infected adults with acute cryptococcal meningitis to describe the range and relative incidence of external ophthalmic signs.

## Methods

The study was nested within the Adjunctive Sertraline for the Treatment of HIV-Associated Cryptococcal Meningitis (ASTRO-CM) trial (ClinicalTrials.gov number: NCT01802385). ASTRO-CM was a phase III randomized controlled trial to evaluate whether adjunctive sertraline improved survival when added to standard amphotericin-based therapy for cryptococcal meningitis. Primary outcome was 18-week survival. We enrolled HIV-infected adults (≥18 years) presenting with cryptococcal meningitis (diagnosed by cerebrospinal fluid (CSF) cryptococcal antigen positivity) to Mulago National Referral Hospital, Kampala or Mbarara Regional Referral Hospital, Uganda between August 2013 and May 2017. Patients were required to be willing to undergo protocol-specified lumbar punctures; and were excluded if they had already received 3 doses of amphotericin B, if they had jaundice or known liver cirrhosis, or were pregnant or currently breastfeeding. Patients were hospitalized for 2 weeks and thereafter seen every 2 weeks as outpatients through 18 weeks.

Standard clinical and examination data were collected as part of the randomized controlled trial. We screened participants for visual disturbance and external ophthalmic signs at baseline and at each follow-up appointment, via clinical history and examination with a simple light source such as a pen torch. Incident ophthalmic symptoms and signs were ascribed by the attending study physician when first observed based on standard clinical case definitions (abnormal signs which persisted were not re-recorded at each follow-up visit).

Analysis was primarily descriptive and was performed using Microsoft Excel® 2016. We summarized the observational data as proportions or medians with inter-quartile ranges (IQR). For the descriptive analysis, we categorized ophthalmic abnormalities into three groups: (i) neurological signs (ii) localized ocular pathology (iii) evidence of concurrent systemic disease.

## Results

We screened 696 adults with HIV-associated cryptococcal meningitis for visual disturbance and external ophthalmic signs. 87.2% of patients presented as a first episode of cryptococcal meningitis, with the remaining 12.8% having had a previous episode. The mean age was 36 years (range 18–70 years), and 60% (421/696) were men. All patients had advanced HIV with a median CD4 count 16 cells/mm ^3^ (IQR 6 - 49) and mean hemoglobin of 11.6 g/dL. Diagnostic lumbar punctures were undertaken on all patients and revealed a median opening pressure of 270 mmHg (IQR 180 – 410), and a median CSF fungal burden of 38,000 cfu/ml (IQR 790 – 236,750).

Overall, 184 participants (26.4%) displayed external ophthalmic signs or symptoms during the study period, with a total of 227 abnormal findings documented ( Table 1). Thirty-eight participants (5.5%) displayed more than one sign or symptom, either concurrently or sequentially (median 2; range 2–4). Ophthalmic signs were diagnosed throughout the study period: at initial assessment (n=80; 11.5%); during inpatient consultation (n=92; 13.2%); and during outpatient consultation (n=65; 9.3%).

**Table 1.** Frequency of external ophthalmic signs and symptoms in Ugandan adults with HIV-associated cryptococcal meningitis. I) Initial assessment (baseline); II) During inpatient stay; III) During subsequent outpatient consultations. Results displayed as raw data and as a percentage of the total study population (N=696). ^a^Reported Visual Disturbance represents the only patient-reported variable; otherwise signs are reported as judged by the clinician. ^b^Not otherwise specified.

Sign or Symptom (N=696)	Baseline n (%)	Inpatient n (%)	Outpatient n (%)	Total n (%)
**Any Ophthalmic Sign or Symptom**	80 (11.5)	92 (13.2)	65 (9.3)	**184 (26.4)**
** Systemic**				
Pallor/Anaemia	12 (1.7)	21 (3.0)	15 (2.2)	**48 (6.9)**
Jaundice/Icterus	2 (0.3)	16 (2.3)	2 (0.3)	**20 (2.9)**
Dehydration/Sunken Eyes	1 (0.1)	3 (0.4)	3 (0.4)	**7 (1.0)**
Periorbital Oedema	4 (0.6)	3 (0.4)	0 (0.0)	**7 (1.0)**
Abnormal Periorbital Skin	4 (0.6)	4 (0.6)	0 (0.0)	**8 (1.1)**
**Total**	23 (3.3)	47 (6.8)	20 (2.9)	**90 (12.9)**
**Ocular**				
*Reported Visual Disturbance ^a^*	*14 (2.0)*	*10 (1.4)*	*8 (1.1)*	***32 (4.6)***
Conjunctivitis/Redness	6 (0.9)	3 (0.4)	13 (1.9)	**22 (3.2)**
Conjunctival Haemorrhage	1 (0.1)	3 (0.4)	1 (0.1)	**5 (0.7)**
Exophthalmos/Proptosis	3 (0.4)	0 (0.0)	0 (0.0)	**3 (0.4)**
Cataract	0 (0.0)	1 (0.1)	0 (0.0)	**1 (0.1)**
**Total**	24 (3.4)	17 (2.4)	22 (3.2)	**63 (9.1)**
**Neurological**				
Abnormal Pupillary Light Reflex				
Unilateral	5 (0.7)	2 (0.3)	2 (0.3)	**9 (1.3)**
Bilateral	2 (0.3)	5 (0.7)	2 (0.3)	**9 (1.3)**
III Palsy				
Unilateral	5 (0.7)	2 (0.3)	2 (0.3)	**9 (1.3)**
Bilateral	0 (0.0)	0 (0.0)	1 (0.1)	**1 (0.1)**
IV Palsy				
Unilateral	1 (0.1)	0 (0.0)	2 (0.3)	**3 (0.4)**
Bilateral	0 (0.0)	0 (0.0)	0 (0.0)	**0 (0.0)**
VI Palsy				
Unilateral	12 (1.7)	4 (0.6)	1 (0.1)	**17 (2.4)**
Bilateral	1 (0.1)	2 (0.3)	1 (0.1)	**4 (0.6)**
VII Palsy				
Unilateral	0 (0.0)	2 (0.3)	1 (0.1)	**3 (0.4)**
Bilateral	0 (0.0)	0 (0.0)	0 (0.0)	**0 (0.0)**
Squint/CN Palsy NOS ^b^	7 (1.0)	2 (0.3)	3 (0.4)	**12 (1.7)**
Conjugate Gaze Palsy				
Lateral	1 (0.1)	2 (0.3)	2 (0.3)	**5 (0.7)**
Vertical	0 (0.0)	1 (0.1)	0 (0.0)	**1 (0.1)**
Nystagmus				
Horizontal	1 (0.1)	0 (0.0)	0 (0.0)	**1 (0.1)**
Vertical	0 (0.0)	1 (0.1)	0 (0.0)	**1 (0.1)**
Bilateral Miosis	1 (0.1)	0 (0.0)	0 (0.0)	**1 (0.1)**
**Total**	36 (5.2)	23 (3.3)	17 (2.4)	**76 (10.9)**

Ninety participants (12.9%) displayed ophthalmic manifestations of systemic disease, with conjunctival pallor the most frequently reported sign (n=48; 6.9%). Sixty-three patients (9.1%) had ocular signs or symptoms, the most common being ‘reported visual disturbance’ (n=32; 4.6%) – a disparate category which grouped all subjective visual deficit in the absence of other objective signs. Conjunctivitis was also frequently noted on examination (n=22; 3.2%). Cranial nerve palsies (III, IV, VI or VII) occurred in 49 patients (7.0%), with the most common being a unilateral VI palsy (n=17; 2.4%). Other neurological symptoms were less commonly seen and included conjugate gaze palsy (n=6), nystagmus (n=2) and bilateral miosis (n=1).

## Conclusions

Ophthalmic signs and symptoms were present in more than a quarter of our cohort of Ugandan adults with HIV-associated cryptococcal meningitis, consistent with previous literature reporting ophthalmic findings to be common in this population ^1, 2, 8, 16, 17^, although heterogeneity in study design should is noted. The complexity of this patient group is demonstrated in the breadth of signs present, both those indicative of systemic disease and those representing concurrent ocular and neurological pathology.

Abnormal examination findings were diagnosed throughout the study period and were equally incident across initial examination, inpatient and outpatient assessment; it is therefore essential that a high clinical vigilance is maintained at every stage of management in these patients.

All reported signs and symptoms were elicited through basic clinical history and examination, requiring only a simple light source such as a pen torch. This demonstrates the potential for identifying a large spectrum of systemic, ocular and neurological disease even in a low-resource setting. The absence of equipment such as Snellen charts in our study may partly explain the low incidence of reported visual disturbance in our population (4.6%), when compared to previously published case series ^2, 8, 16, 17^. The apparently low incidence of neurological signs (10.9%) is likely to be attributable in part to the effect of protocol-specified lumbar punctures on intracranial pressure.

Of note, signs of systemic disease such as conjunctival pallor (6.9%) and scleral icterus (2.9%) were particularly incident in our population. Anaemia is a common feature of advanced HIV disease and a side-effect of amphotericin B-based induction therapy, with increased mortality in patients with anaemia at baseline ^18^. Hepatotoxicity is less common, but also well-described for both amphotericin B and fluconazole, as well as several commonly-used antiretroviral (ARV) drugs such as nevirapine and efavirenz. Jaundice may signal concomitant infection with chronic hepatitis C virus (HCV) or hepatitis B virus (HBV), as well as other opportunistic infections such as cytomegalovirus (CMV) and tuberculosis (TB). Simple ophthalmic examination can provide a mechanism for identifying these potentially complicating issues - both prior to and during anti-fungal treatment - and prompt further investigation.

Our data afford several advantages compared to previous case series. Firstly, the size of our study population (N=696) is considerably larger than those previously examined, allowing for a more comprehensive description of signs and symptoms and their relative incidence. Secondly, we assessed patients regularly across a range of time-points. Systematic screening at baseline allowed for identification of early ophthalmic signs, with prolonged (18-week) follow-up ensuring late manifestations were also captured. Finally, the simplicity of clinical examination allowed a wide range of presentation severities to be assessed, in contrast to previous studies limited to co-operative patients able to consent to comprehensive ophthalmological assessments ^2^.

However, we recognise several limitations to our study. Firstly, although visual disturbances were systematically screened for, due to the nested nature of the study formal visual acuity testing was not performed, and therefore it is likely that we underestimated the proportion of patients with objective visual disturbance. Secondly, neither slit lamp examination nor indirect ophthalmoscopy was performed and therefore we are unable to comprehensively report on the prevalence of asymptomatic anterior chamber or retinal pathology in our cohort. Thirdly, in most cases, no attempt was made to establish aetiology of reported visual disturbance. The use of dilated indirect ophthalmoscopy in conjunction with intracranial pressure measurement and imaging, would have aided in dividing ocular from neurological pathology. Mechanisms for visual loss have been discussed elsewhere ^2, 19^. In addition, we did not extract data regarding co-morbidities or medications with ophthalmic consequences, such as diabetes mellitus or ethambutol. Finally, our study was underpowered to detect an association between visual disturbance, cryptococcal disease severity and outcome.

Currently, limited published data exist describing ophthalmic features observed in HIV-associated cryptococcal cohorts. Prospective study – including routine slit lamp and indirect ophthalmoscopic examination - is urgently required into the correlation of reported visual disturbance with objective signs, in order to further clarify the underlying mechanisms and to guide effective diagnosis, follow-up and management.

## Consent

Ugandan (MREC 429) and Minnesota (1304M31361) Institutional Review Boards approved the ASTRO-CM trial protocol. All participants (or a surrogate, should the former demonstrate altered mental status or otherwise lack capacity) provided written informed consent.

## Data availability

The database contains individual level data and as such is not available through an open-access data repository. The database is stored on a secure server at University of Minnesota. Researchers interested in accessing the data can contact the corresponding author (RRA; rachelatherton@doctors.org.uk), the last author (DRB; boulw001@umn.edu) or the Division of Biostatistics at the University of Minnesota ( sph-ask@umn.edu). Data access will be granted to active researchers in the field with the agreement of the authors.
