# Supplementary material for: Ophthalmic signs in Ugandan adults with HIV-associated cryptococcal meningitis: A nested analysis of the ASTRO-CM cohort
Source: Wellcome Open Res. 2018 Oct 12;3:80. Originally published 2018 Jun 27. [Version 2] doi: 10.12688/wellcomeopenres.14666.2 (PMC6178913; doi:10.12688/wellcomeopenres.14666.2)
Supplement: Peer review history

# Peer review: Referee response for version 2

**DOI:** 10.21956/wellcomeopenres.16207.r34063

**Published:** 2018-10-22

**Authors:** Héctor Pérez, Emilio Dodds

**Affiliations:** 1 Fernández Hospital, Buenos Aires, Argentina; 2 Infectious Diseases Division , Fernández Hospital, Buenos Aires, Argentina

We have read this submission. We believe that we have an appropriate level of expertise to confirm that it is of an acceptable scientific standard.

# Peer review: Referee response for version 1

**DOI:** 10.21956/wellcomeopenres.15968.r34000

**Published:** 2018-10-08

**Authors:** Héctor Pérez, Emilio Dodds

**Affiliations:** 1 Infectious Diseases Division , Fernández Hospital, Buenos Aires, Argentina; 2 Fernández Hospital, Buenos Aires, Argentina

This work was evaluated jointly, with Dr. Emilio Dodds, who is a reference of the ophthalmology service and consultant of the infectious diseases division of the Juan A. Fernandez Hospital, in the city of Buenos Aires, with extensive experience in diagnostic, treatment and monitoring of more than four thousand patients since 1982, with multiple studies published, continuing their work for more than thirty-five years, and training resident doctors, as well as dictating the subject in the undergraduate program. These are our conclusions.

 Overall it is a nice study with a big population, however the ophthalmic study is limited and the authors clearly state that condition. Some comments: 

1. The title is misleading: I would say external ophthalmic findings instead of ophthalmic signs, since this is exactly what they studied and they did not look for the whole ophthalmic signs.
2. To clarify I would add external ophthalmic signs every time they mention ophthalmic signs (abstract, row 10; Background, last row; Methods, second paragraph, 3 row; Results, 2 row)
3. Conclusion: ophthalmic signs and symptoms were present in more than a quarter of our cohort…consistent with previous reports (references 1, 2, 8, 16,17). These studies are not comparable since they are looking at different things: external findings and not the whole signs.
4. Page 5, first column, last paragraph, row 5: they say the underestimate number of patients with visual disturbances but they could also overestimate it because they are not checking for refractive errors.

We have read this submission. We believe that we have an appropriate level of expertise to confirm that it is of an acceptable scientific standard.

# Peer review: Referee response for version 1

**DOI:** 10.21956/wellcomeopenres.15968.r33421

**Published:** 2018-07-02

**Authors:** Mosepele Mosepele

**Affiliations:** 1 Botswana Harvard AIDS Institute Partnership (BHP), Gaborone, Botswana; 2 Department of Internal Medicine, Faculty of Medicine, University of Botswana, Gaborone, Botswana

Study question, setting & population, study measures, data analysis, data reporting & interpretation are clearly stated. In this ophthalmic complications study nested within a CM trial, the investigators described ophthalmic complications among 696 HIV-CM adults in Uganda. At any point in time (at admission, during admission, post-discharge), rate of ophthalmic complications ranged between 9 and 13%. Clinically significant findings such as subjective decrease in visual acuity were however, uncommon.

 Suggestions for data reporting:

1. Would have been useful to "trend" out the TYPE(S) of ophthalmic complications over time (even though the total frequencies were similar throughout follow-up). For instance, give baseline median haemoglobin of 11g/dl, the reported 48 participants may indicate complications of Amphotericin (late in course of treatment) vs at presentation. A figure or bar chart or some other graphical representation of major findings at each setting: at admission vs during admission vs post-discharge may be informative re the type of further care/evaluation that could be recommended for patients (systemic disease will be for the treating clinician) while ocular/neuro symptoms may indicate need for eye clinic referral.
2. In this setting, proportion of patients started on Ethambutol (or even anti-tubercuolous therapy) containing antimicrobial therapy should be reported if available - or these limitation discussed as Ethambutol may cause decrease in visual acuity.

Conclusions:

1. Neurologic complications seem very low. Discussing the trial effect, e.g., intensive CSF drainage vs opening pressure monitoring as per standard of care, should be discussed.
2. Evaluation of other conditions that may results in ophthalmic conditions such as Diabetes, etc were not evaluated. This should be discussed in the study limitations section.

I have read this submission. I believe that I have an appropriate level of expertise to confirm that it is of an acceptable scientific standard.
